# Olfactomedin 4 as a novel loop of Henle‐specific acute kidney injury biomarker

**DOI:** 10.14814/phy2.15453

**Published:** 2022-09-19

**Authors:** Denise C. Hasson, Kelli Krallman, Katherine VanDenHeuvel, Shina Menon, Giovanna Piraino, Prasad Devarajan, Stuart L. Goldstein, Matthew N. Alder

**Affiliations:** ^1^ Division of Critical Care Medicine Cincinnati Children's Hospital Medical Center Cincinnati Ohio USA; ^2^ Division of Nephrology and Hypertension Cincinnati Children's Hospital Medical Center Cincinnati Ohio USA; ^3^ Division of Pathology and Laboratory Medicine Cincinnati Children's Hospital Medical Center Cincinnati Ohio USA; ^4^ Division of Nephrology and Hypertension Seattle Children's Hospital Seattle Washington USA; ^5^ Department of Pediatrics University of Cincinnati College of Medicine Cincinnati Ohio USA

**Keywords:** acute kidney injury, loop of Henle, olfactomedin 4, septic AKI, urine biomarker

## Abstract

Acute kidney injury (AKI) is associated with morbidity and mortality. Urinary biomarkers may disentangle its clinical heterogeneity. Olfactomedin 4 (OLFM4) is a secreted glycoprotein expressed in stressed neutrophils and epithelial cells. In septic mice, OLFM4 expression localized to the kidney's loop of Henle (LOH) and was detectable in the urine. We hypothesized that urine OLFM4 (uOLFM4) will be increased in patients with AKI and sepsis. Urine from critically ill pediatric patients was obtained from a prospective study based on AKI and sepsis status. uOLFM4 was quantified with a Luminex immunoassay. AKI was defined by KDIGO severe criteria. Sepsis status was extracted from the medical record based on admission diagnosis. Immunofluorescence on pediatric kidney biopsies was performed with NKCC2, uromodulin and OLFM4 specific antibodies. Eight patients had no sepsis, no AKI; 7 had no sepsis but did have AKI; 10 had sepsis, no AKI; 11 had sepsis and AKI. Patients with AKI had increased uOLFM4 compared to no/stage 1 AKI (*p* = 0.044). Those with sepsis had increased uOLFM4 compared to no sepsis (*p* = 0.026). uOLFM4 and NGAL were correlated (*r* ^2^ 0.59, 95% CI 0.304–0.773, *p* = 0.002), but some patients had high uOLFM4 and low NGAL, and vice versa. Immunofluorescence on kidney biopsies demonstrated OLFM4 colocalization with NKCC2 and uromodulin, suggesting expression in the thick ascending LOH (TALH). We conclude that AKI and sepsis are associated with increased uOLFM4. uOLFM4 and NGAL correlated in many patients, but was poor in others, suggesting these markers may differentiate AKI subgroups. Given OLFM4 colocalization to human TALH, we propose OLFM4 may be a LOH‐specific AKI biomarker.

## Graphical abstract

Acute kidney injury is a common and heterogeneous problem that would benefit from biomarkers that allow improved prognostication and ultimately treatment options. We show that Olfactomedin 4 (OLFM4) is present in human kidney following injury. Immunohistochemistry suggests OLFM4 co‐localizes with NKCC2 and uromodulin and may serve as an injury marker for the loop of Henle.

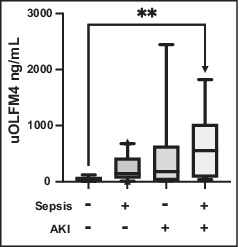

## 1 INTRODUCTION

Acute kidney injury (AKI) occurs frequently in critically ill adults and children (Hoste et al., 2006; Kaddourah et al., 2017; Uchino et al., 2005), and it is associated with greater hospital costs, longer durations of high‐risk interventions, greater intensive care unit (ICU) and hospital lengths of stay (LOS), and mortality (Bagshaw et al., 2006; Korkeila et al., 2000; Manns et al., 2003; Morgera et al., 2002). It is recognized that not all AKI is the same. Different etiologies, severity, duration, and timing of AKI can impact patient‐specific outcomes (Basu et al., 2021; Gist et al., 2022).

Novel AKI biomarkers have the potential to disentangle the clinical heterogeneity and help clinicians better understand the pathophysiology of AKI. The 23rd Acute Disease Quality Initiative (ADQI) conference focused on AKI diagnostics and emphasized the importance of finding biomarkers that will refine AKI diagnosis based on pathophysiologic process, etiology, and location of injury (ADQI 23 figure 2) (Ostermann et al., 2020). Of the 23 different biomarkers reviewed, none were expressed specifically by the loop of Henle (LOH). In addition to protein biomarkers, specific tests of renal tubular health can predict poor AKI outcomes. One of these, the furosemide stress test, can predict progression to stage III AKI (Chawla et al., 2013) and future receipt of renal replacement therapy better than any novel biomarker in adults (Koyner et al., 2015; Lumlertgul et al., 2018) and children (Gist et al., 2021; Kakajiwala et al., 2017).

Olfactomedin 4 (OLFM4) is a secreted glycoprotein expressed in mature neutrophils and epithelial cells in prostate and gut epithelium following stress. In states of normal health, only about ~25% of human neutrophils express OLFM4 (Clemmensen et al., 2012); however, it is one of the most upregulated genes in the peripheral blood of patients with sepsis (Wong et al., 2009). In pediatric patients with septic shock, increased OLFM4 mRNA transcription, plasma protein levels, and a greater percentage of OLFM4 positive neutrophils are independently associated with multiorgan failure and death (Alder et al., 2019). OLFM4 null mice are protected from death in sepsis models, suggesting its role in the immune response (Liu et al., 2010; Liu et al., 2013).

Our group recently found that wild type murine pups challenged with sepsis showed increased OLFM4 expression that localized to the kidney, specifically to the LOH. Healthy control animals do not express OLFM4; only following septic challenge and renal injury was OLFM4 expression detected in the LOH and in the urine. OLFM4 null murine pups with sepsis were protected from renal cell apoptosis and plasma creatinine elevation seen in their wild‐type septic counterparts (Stark et al., 2020). Given this renal expression of OLFM4 and association with change in kidney function, we hypothesized that urine OLFM4 (uOLFM4) will be increased in pediatric patients with AKI and sepsis, and that it will localize to the thick ascending LOH (TALH) in pediatric kidney tissue.

## 2 MATERIALS AND METHODS

### 2.1 Patient enrollment and urine collection

Patients were initially enrolled in the “AKI in Children Expected by Renal angina and Urinary Biomarkers” (AKI‐CHERUB, NCT01735162) study at Cincinnati Children's Hospital Medical Center (CCHMC; Menon et al., 2016). Briefly, this was a prospective observational study conducted from September 2012 to March 2014 in the CCHMC pediatric ICU. All children admitted from ages 3 months to 25 years with a urinary catheter and anticipated discharge >48 hours from pediatric ICU admission were enrolled, excluding patients with end‐stage renal disease (ESRD) and immediately post‐renal transplant. Urine samples were collected, centrifuged, and stored at −80°C. For the purpose of this study, day 1 urine samples were used for OLFM4 analysis. Samples were chosen based on AKI and sepsis status. Sepsis diagnoses were extracted from the electronic medical record and categorized as yes/no based on ICU admission diagnosis in the ICU note. Demographic and lab data were extracted from the medical record. Neutrophil gelatinase associated lipocalin (NGAL) was assayed using a human‐specific commercially available enzyme‐linked immunosorbent assay (ELISA, AntibodyShop, Grusbakken, Denmark) for the CHERUB study (Menon et al., 2016). The original study was approved by the CCHMC Institutional Review Board (IRB) with waiver of the need for informed consent. Study procedures were in accordance with the ethical standards of the responsible committee on human experimentation (institutional and national) and with the Helsinki Declaration of 1975, as revised in 2000.

### 2.2 AKI staging

AKI staging was determined per the original AKI‐CHERUB study protocol. Patients with AKI met criteria for severe, persistent AKI by Kidney Disease Improving Global Outcomes (KDIGO) serum creatinine criteria, or a >2 times change in serum creatinine from baseline present on day 3 of pediatric ICU admission (Kellum et al., 2014).

### 2.3 Urinary OLFM4 measurement

A custom kit specific for human OLFM4 was developed by EMD Millipore. Urine OLFM4 concentration was measured following the kit protocol and assayed on a Luminex 200 Instrument. Western blots for OLFM4 were done using standard protocols with OLFM4 detected using OLFM4‐PA5‐85041 (Thermo Scientific).

### 2.4 Immunohistochemistry and immunofluorescence

Human kidney block sections were selected by a pathologist who specializes in pediatric kidney disease. For uromodulin staining, four control sections were chosen from patients with Wilms' tumor or pathology not expected to have injury to the tubules, acknowledging that biopsies are not performed on pediatric patients with healthy kidneys. Four sections with acute tubular necrosis (ATN), three sections from patients with a renal transplant and tubular injury, and two sections from patients with chronic/ongoing tubular injury were prepared. Slides were deparaffinized and rehydrated with successive xylene, ethanol, and aqueous baths. Antigen retrieval was done by heating in citrate buffer. Endogenous biotin was blocked with Avidin biotin blocking kit (Biocare Medical) and peroxidase blocked with Bloxall (Vector Laboratories). Slides were then stained with anti‐human OLFM4‐PA5‐85041 and Uromodulin‐PA5‐46959 (Thermo Scientific) followed by secondary antibodies. A similar protocol was used for staining with anti NKCC2 (Abcam ab240542) except that only two sections from patients with AKI were stained. Images were taken on a Nikon TiE SpectraX microscope. The CCHMC IRB approved use of human kidney tissue for the purpose of this study. Furthermore, given the finite nature of the resource collection, the Department of Pathology approved the request for specimen use after Biobank Biospecimen and Data Request Form was completed and evaluated.

### 2.5 Statistics

All statistical analyses were performed using SigmaStat (Systat Software) and GraphPad‐Prism. As all groups were non‐normally distributed, comparisons between different groups were performed using the Mann–Whitney *U* test, Kruskal Wallis test, and Spearman correlation. Data is presented as box and whisker plots with upper and lower whiskers representing the 90th and 10th percental. An area under the receiver operating curve (AUC‐ROC) analysis was performed to assess whether OLFM4 could have discriminatory ability to differentiate patients with AKI from those without AKI. A cutoff *p*‐value of 0.05 was used to establish statistical significance.

## 3 RESULTS

### 3.1 Patient demographics

We analyzed urine from 8 patients without sepsis or AKI, 7 without sepsis but with AKI, 10 with sepsis but without AKI, and 11 with sepsis and AKI (Table 1). The median age in each group ranged from 4.1 to 12.3 years, 50%–90% of each group was female except the no sepsis/AKI group that had only 1 female, and the majority of patients were of white ethnicity. Neither median mechanical ventilation days, median ICU days, nor mean baseline serum creatinine levels differed between groups. The only patient who died had sepsis but no AKI, and 1 patient in each AKI group required renal replacement therapy (RRT).

**TABLE 1:** Demographics and outcomes by patient group

	No sepsis no AKI (*n* = 8)	No sepsis yes AKI (*n* = 7)	Yes sepsis no AKI (*n* = 10)	Yes sepsis yes AKI (*n* = 11)	*p* value
Age, years, mean (SD)	6.7 (7.3)	10.2 (6.6)	10.4 (5.6)	8.4 (4.9)	0.57
Female (%)	4 (50)	1 (14)	9 (90)	7 (63)	
White	6	5	9	10	
Black	1	2	1	0	
Hispanic	1	0	0	1	
MV days, median (IQR)	0.5 (0.0–1.0)	3 (2.0–13.0)	2 (0.0–6.3)	2 (0.0–4.0)	0.209
ICU days, median (IQR)	4.5 (2.0–10.5)	4 (2.0–16.0)	6.5 (2.8–16.8)	7 (4.0–13.0)	0.553
Hospital days, median (IQR)	11.5 (5.3–45.5)	8 (5.0–24.0)	26 (6.8–36.5)	11(8.0–58.0)	0.621
Need for RRT	0	0	1	1	
ICU Mortality	0	0	1	0	
Baseline SCr, mean (SD)	0.43 (0.23)	0.51 (0.22)	0.44 (0.14)	0.37 (0.15)	0.473

Abbreviations: ICU, intensive care unit; MV, mechanical ventilation; RRT, renal replacement therapy; SCr, serum creatinine.

### 3.2 Patients with AKI had elevated urine OLFM4 and NGAL compared to those without AKI

**FIGURE 1:**
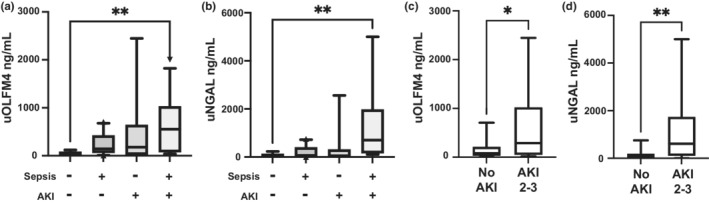
Urine OLFM4 and NGAL is increased in patients following severe AKI. (a) Box and whisker plot showing urine OLFM4 levels from patients in all 4 groups, no sepsis and no AKI (*n* = 8), sepsis and no AKI (*n* = 10), AKI and no sepsis (*n* = 7), and septic AKI (*n* = 11), *p* = 0.0342. (b) Box and whisker plot showing urine NGAL levels from patients in the same 4 groups (*n* = 8, 10, 6, and 11 respectively), *p* = 0.0109 (c) uOLFM4 levels and (d) uNGAL levels in patients with no/stage 1 AKI vs stage 2–3 severe AKI (*n* = 17‐18/group), *p* = 0.0435 and 0.0027, respectively. *p < 0.05. **p < 0.005. uOLFM4, urine olfactomedin 4, uNGAL, urine neutrophil gelatinase associated lipocalin.

We assessed uOLFM4 concentration in all four groups of patients with and without sepsis and AKI (Figure 1a). There was a trend toward higher uOLFM4 in each group with AKI, but this only reached statistical significance when comparing patients without sepsis or AKI to those with septic AKI (*p* = 0.028). We performed the same analysis with urine NGAL, which produced similar results (Figure 1b). Patients with septic AKI had higher NGAL concentrations than patients without sepsis or AKI (*p* = 0.007). To verify that our ELISA assay was indeed detecting full length OLFM4 in the urine, we performed a Western blot for OLFM4 from human urine from patients with low, medium, and high concentration of OLFM4 based on ELISA results. Bands on the blots corresponded to the predicted molecular weight for OLFM4 and increased in intensity from low to high concentration (Figure S1).

We analyzed uOLFM4 from all 18 patients with AKI and compared them to the 18 patients with stage 1/no AKI. Patients with AKI had higher uOLFM4 (median 288.4 [IQR 48.9–1023] versus 81.7 ng/ml [IQR 27.66–211.4], *p* = 0.044, Figure 1c). These results are similar to urine NGAL levels seen in those with AKI compared to those without (*n* = 18 and 17, respectively, median 612.4 [IQR 109–1738] versus 50.8 ng/ml [IQR 4.1–185.6], *p* = 0.003, Figure 1d).

### 3.3 Patients with sepsis had elevated urine OLFM4 and urine NGAL

**FIGURE 2:**
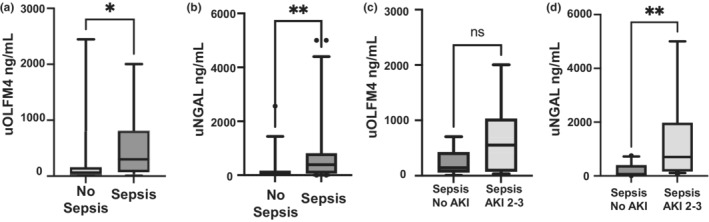
Urine OLFM4 and urine NGAL levels in sepsis. Box and whisker plots showing (a) urine OLFM4 levels and (b) urine NGAL levels in patients without vs with sepsis (*n* = 14–15 and 21, respectively), *p* = 0.0255 and *p* = 0.0095. Comparisons between (c) urine OLFM4 levels and (d) urine NGAL levels in septic patients with no AKI vs with septic AKI (*n* = 10 and 11, respectively), *p* = 0.1321 and *p* = 0.0027. *p < 0.05. **p < 0.005. uOLFM4, urine olfactomedin 4, uNGAL, urine neutrophil gelatinase associated lipocalin.

We compared urine from 21 patients with sepsis to 15 without sepsis. uOLFM4 was elevated in septic patients (median 302.1 [IQR 73.8–811.4] vs 59.06 ng/ml [IQR 21.84–178], *p* = 0.026, Figure 2a), as was urine NGAL (*n* = 21 and 14, respectively, median 394.5 [IQR 69.4–808.3] vs 47.79 ng/ml [IQR 0.32–156.3], *p* = 0.0095, Figure 2b). Thus, both protein concentrations are elevated in the urine during sepsis.

To further assess the effect of sepsis and AKI on these urinary proteins, we compared just those patients with sepsis. uOLFM4 was not significantly higher in patients with septic AKI (*n* = 11) compared to sepsis and no AKI (*n* = 10, median 552.5 [IQR, 75.2–1030] versus 143.2 ng/ml [IQR 61.4–424.4], *p* = 0.132, Figure 2c). Urine NGAL was able to differentiate patients with septic AKI compared to sepsis and no AKI (median 701.6 [IQR 164.3–1978] vs 69.4 ng/ml [IQR 4.25–402.4], *p* = 0.0027, Figure 2d).

### 3.4 Correlation between uOLFM4 and NGAL

**FIGURE 3:**
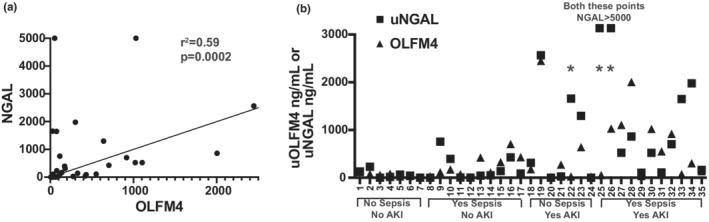
Correlation between uOLFM4 and uNGAL values. (a) Spearman correlation between uOLFM4 and uNGAL levels by individual patient. (b) Individual patient levels of uOLFM4 (triangles) and uNGAL (boxes), grouped by AKI and sepsis status. Two patients had uNGAL levels >5000, shown at the limit of the y‐axis. * indicates patients with disparate uOLFM4 and uNGAL values. uOLFM4‐urine olfactomedin4. uNGAL, urine neutrophil gelatinase associated lipocalin.

**FIGURE 4:**
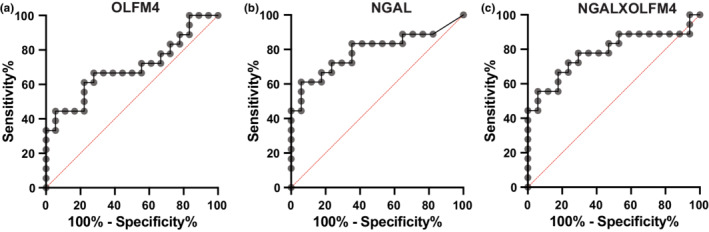
Receiver operating curve showing ability of (a) urine OLFM4, (b) urine NGAL, and (c) the product of both levels to discriminate between patients without vs with AKI.

We observed correlation between urine NGAL and uOLFM4 (*r* ^2^ 0.59, 95% CI 0.304–0.773, *p* = 0.002, Figure 3a); however, there are some patients with high NGAL and low uOLFM4 and others with high uOLFM4 and low NGAL (Figure 3b, asterisk). Finally, we compared the receiver operating curves for uOLFM4 and NGAL for predicting AKI. uOLFM4 had moderate ability to discriminate those with AKI from those without, with an AUC of 0.69 (95% CI 0.52–0.87; Figure 4a). NGAL had an AUC of 0.79 (95% CI 0.63–0.94; Figure 4b). We multiplied the two levels together to see if the product improved AUC for predicting kidney injury. The median of this product in patients with AKI was 196,988.2 [IQR 28,022.3–632,124.1] versus 5502.8 ng/ml [IQR 320.6–37,904.1] in those without AKI (*p* = 0.0034). The AUC for the product of OLFM4 and NGAL for detecting stage 2–3 AKI was 0.78 (95% CI 0.63–0.94; Figure 4c), similar to that of NGAL alone (AUC 0.79, 95% CI 0.63–0.94).

### 3.5 OLFM4 localizes to human TALH

Two of four control samples had no detectable OLFM4 staining and two had rare OLFM4 expression, all of which colocalized with uromodulin. From the nine samples with AKI, eight had moderate amounts of OLFM4 signal, almost all of which colocalized with uromodulin (Figure S2). Occasionally OLFM4 staining appeared to colocalize with cells; however, a lot of it appeared intraluminal, especially when localizing with uromodulin. Not all LOH cells produced OLFM4, as staining for OLFM4 was scattered throughout. In one patient with ATN of unknown etiology, OLFM4 did not always colocalize with uromodulin. Taken together, the vast majority of OLFM4 staining colocalized with uromodulin, suggesting OLFM4 expression comes from the LOH.

**FIGURE 5:**
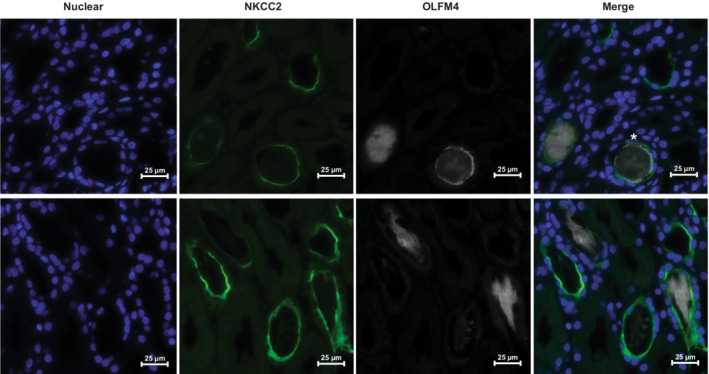
Immunofluorescence from a human kidney biopsy from a patient with AKI. Dapi was used to identify nuclei. Green fluorescence can be seen on the luminal surface of some tubules marking expression of NKCC2. White fluorescence represents OLFM4 expression. Most OLFM4 staining is intraluminal with rare luminal surface expression (*).

To further attempt to localize OLFM4 expression we performed dual staining for Na‐K‐CL (NKCC2), the receptor targeted by the loop diuretic furosemide, and OLFM4 from two patients with AKI (Figure 5). Staining for NKCC2 could be seen at the expected location on the luminal surface of some of the tubules (green fluorescence). OLFM4 staining was again appreciated predominately in within tubular lumen, with rare colocalization that appeared surface expression (Figure 5 top row, *). Not all tubules with OLFM4 within the lumen could be identified by NKCC2 staining.

## 4 DISCUSSION

The clinical management and study of potential therapies for AKI have been hindered by imprecise, late, and inaccurate biomarkers used to diagnose this condition. There is a great deal of heterogeneity within patients with AKI that arises from injury to different regions of the nephron, different mechanisms of injury, and different genetic backgrounds. Herein, we have identified a protein that colocalizes with the TALH region of the kidney and that correlates with creatinine‐diagnosed AKI; thus, we propose this may be a LOH‐specific AKI biomarker. An array of urinary biomarkers to help localize renal injury may assist in the deconvolution of heterogeneity in AKI.

Tamm‐Horsfall protein, or uromodulin, has been the main protein biomarker to date associated with the TALH (El‐Achkar et al., 2013; Wen & Parikh, 2021). Previous work has shown a negative correlation between serum uromodulin and AKI in conditions like ANCA‐associated vasculitis (Tachibana et al., 2019) and ischemia–reperfusion injury (IRI; El‐Achkar et al., 2013), and between urine uromodulin and AKI in diabetes (Chakraborty et al., 2004) and patients undergoing cardiac surgery (Zhang et al., 2022). Osteopontin, a bone phosphoprotein, is produced predominantly in the TALH but also in the distal convoluted tubule. In states of injury, its expression is upregulated, which is why it has become a novel AKI biomarker. However, this upregulation is not tubule‐segment specific and even occurs in the glomerulus (Xie et al., 2001). Only in the past year, Chorley et al attempted to remedy the lack of nephron segment‐specific biomarkers by using differential expression of urinary micro RNA (miRNA) to identify renal damage in a nephrotoxin‐induced kidney injury model in rats. In this novel study, they found miR‐221‐3p, miR‐222‐3p, and miR‐210‐3p were increased in the urine of rats treated with TALH‐specific nephrotoxic agents, suggesting these could be future LOH‐specific biomarkers (Chorley et al., 2021). This technology has many steps prior to its implementation in clinical use.

We found a moderate correlation between OLFM4 and NGAL, as both are neutrophil granule proteins secreted by activated neutrophils and expressed by the nephron following injury. We could not prove OLFM4 specific origin, as do not know whether these proteins in the urine came from glomerular filtering of systemically produced OLFM4 vs tubular production. In bone marrow transplant experiments performed in septic mice, Stark et al found that mice null for OLFM4 in their blood neutrophils still produced OLFM4 locally by tubular cells (Stark et al., 2020). Because of this, we believe that the colocalization we observed is not related to leukocyte migration or selective filtration but is reflective of de novo intrinsic kidney production. We almost exclusively localized OLFM4 with uromodulin, but the OLFM4 was predominantly intraluminal, bringing up the possibility that OLFM4 is trapped within uromodulin‐based casts. Further staining attempted to localize OLFM4 expression with NKCC2 which showed rare co‐expression, however, at the time these clinical biopsies were collected, OLFM4 was primarily within the tubules. It is quite possible that following secretion into the tubular lumen, OLFM4 migrates down toward the collecting duct, which would explain why not all OLFM4 within the lumen colocalized with NKCC2. It is similarly known that tubular cells upregulate production of NGAL; this production occurs non‐specifically in the distal convoluted tubules, collecting ducts, and even to some extent the LOH, and there is reabsorption in the proximal tubules, which limits its anatomic specificity (Singer et al., 2013).

In this small study, OLFM4 was still not as sensitive nor specific for AKI compared to NGAL, and we do not believe it should be used alone as an AKI diagnostic biomarker. However, we believe there is potential clinical utility in using both urinary biomarkers together. By targeting different anatomic locations, it is possible that a panel of OLFM4 and NGAL may provide greater information as to the specific nature of the kidney injury than either alone. Despite our statistically significant correlation between these two biomarkers, the r of 0.59 leaves much room for parsing out of differences between the biomarkers, and there are clearly patients who have elevation of one but not the other. Because there are some patients with high OLFM4 and low NGAL and vice versa, we wondered if the product of both levels would improve the sensitivity for detecting AKI, and it did not. It does appear that NGAL is generally higher than OLFM4, except in patients with septic AKI. It is possible that the outliers resulting in significantly higher NGAL than OLFM4 could be from leukocyturia in patients with urinary tract infections, a population in whom NGAL is known to be unreliable (Cullen et al., 2012; Yilmaz et al., 2009).

Anatomic localization of biomarkers is important given the unique roles each segment plays in renal function. The main role of the TALH in clinical practice is its urinary concentrating abilities and pharmacologic manipulation with loop diuretics to induce natriuresis. In the past decade, the ability of tubular response to standardized doses of furosemide, known as the “furosemide stress test,” has shown ability to predict progression to advanced AKI stages (Chawla et al., 2013) and receipt of renal replacement therapy (Lumlertgul et al., 2018) better than novel damage biomarkers (Koyner et al., 2015). However, this test is only effective when the patient is intravascularly replete, and many providers do not feel comfortable giving furosemide when a patient is hemodynamically unstable. Therefore, the timing of furosemide administration is often when the patient has incurred significant and irreversible renal damage and is too late for predictive utility. A urinary biomarker that elevates early in the disease process and that predicts furosemide responsiveness would give clinicians a tool to predict who may benefit from RRT without having to wait for hemodynamic stability. Only 22 of 36 of our patients received furosemide, on average 2.8 days after PICU admission, and only six patients received 1 mg/kg IV or greater doses. Therefore, we were unable to assess whether uOLFM4 levels could predict a positive or negative furosemide stress test. Ideal future studies would analyze urine levels of OLFM4 at 12–24 hours after kidney insult and administer higher furosemide doses earlier in the ICU course.

Another possible application of OLFM4 as a novel biomarker may be in the diagnosis of septic AKI. Sepsis is the most common etiology of AKI in the ICU, responsible for 40%–50% of AKI in critically ill adults and children (Bagshaw et al., 2007; Bagshaw et al., 2008; Hoste et al., 2003). Septic AKI is associated with increased fluid overload; greater oliguria; longer duration of mechanical ventilation, ICU, and hospital LOS (Bagshaw et al., 2008; Bouchard et al., 2015; Hoste et al., 2003). Additionally, septic AKI confers a 20%–30% higher mortality in children than AKI of other etiologies (Basu et al., 2018; Duzova et al., 2010). Again, this etiology‐specific AKI is especially difficult to diagnose. Increased fluid overload in this population dilutes serum creatinine, making this functional biomarker even less reliable. Additionally, less is known about novel biomarkers in this patient population. One of the main limitations of NGAL is its systemic elevation in inflammatory states, driven by IL‐6 mediated hepatocyte production (Skrypnyk et al., 2020). An ideal marker of septic AKI would not be elevated by sepsis alone but would rise when a septic patient gets AKI. While further studies with larger cohorts of patients need to be done, our findings of increased OLFM4 in patients with septic AKI may be promising. Having a septic AKI‐specific biomarker would allow clinicians to target this particularly high‐risk cohort and implement AKI mitigation protocols earlier.

Strengths of this study are the novel discovery of OLFM4 as a biomarker of AKI that is released into and easily measured in very small aliquots of urine. We have further supported what was found in an experimental mouse model of sepsis, namely that OLFM4 expression colocalizes to the TALH in human kidney tissue, with healthy tissue rarely expressing OLFM4, and injury increasing its expression. The main limitations of this pilot study are the very small number of patients analyzed, and the further need to better classify sepsis status. The sepsis syndrome is very difficult to diagnose clinically; using diagnoses from the health record could have caused misclassification of patients. However, despite this small number, statistical significance was achieved in many of the analyses, pointing to the strength of this signal. Another limitation is not knowing whether patients in our small cohort had urinary tract infections or leukocyturia for other reasons; this would skew NGAL concentrations as mentioned above and may account for a lot of the differences between OLFM4 and NGAL. We do not know the impact of leukocyturia on OLFM4. Future studies will evaluate the association between urinary OLFM4, AKI, and sepsis from a larger, prospectively recruited pediatric ICU cohort, with a subgroup analysis focusing on septic AKI. Our OLFM4 localization experiments are limited by the fact that kidney biopsies are rarely collected for AKI and the few biopsies we were able to examine may not be ideally timed for OLFM4 expression.

In conclusion, OLFM4 is elevated in the urine of patients with AKI and sepsis, and there was a correlation between uOLFM4 and NGAL. Given OLFM4 colocalization to human TALH and NKCC2, we propose OLFM4 may be a TALH‐specific AKI biomarker. Future studies will look to corroborate these findings prospectively, focusing on septic AKI, and evaluating whether OLFM4 will be able to predict response to a standardized furosemide dose in patients with kidney injury.

## AUTHOR CONTRIBUTIONS

Denise C. Hasson and Matthew N. Alder performed data analysis, interpretation, drafted, and revised the manuscript. Matthew N. Alder was responsible for conception of the study, with input from Denise C. Hasson, Prasad Devarajan, Stuart L. Goldstein. Kelli Krallman and Shina Menon provided intellectual content of critical importance and aided in editing/revising. Katherine VanDenHeuvel was responsible for pulling pathology specimens, interpreting immunofluorescence, and editing the manuscript. Giovanna Piaino assisted with experiments and interpreting data. All authors approved of the final version to be published.

## FUNDING INFORMATION

MN Alder: NIH K08GM12498.

## CONFLICT OF INTEREST

Stuart L Goldstein reports receiving personal fees from Baxter Healthcare, BioPorto Inc., CHF Solutions, Fresenius, MediBeacon, and Medtronic. Prasad Devarajan is a co‐inventor on submitted patents for the use of NGAL as a biomarker for kidney injury.

## Supporting information


Figure S1

Figure S2
