# Supplementary material for: Olfactomedin 4 as a novel loop of Henle‐specific acute kidney injury biomarker
Source: Physiol Rep. 2022 Sep 19;10(18):e15453. doi: 10.14814/phy2.15453 (PMC9483618; doi:10.14814/phy2.15453)
Supplement: Supplementary file 1 — Figure S1 [file PHY2-10-e15453-s002.pdf]

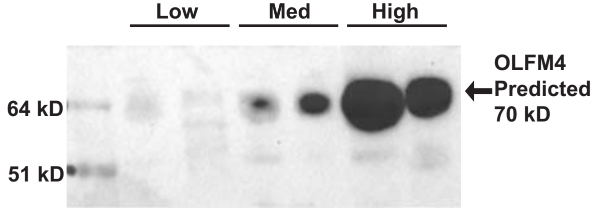

Supplementary Figure 1. Western Blot Showing OLFM4 in Human Urine. Blot using anti-OLFM4 polyclonal antibody from patients with very low (low), medium (med), and high (high) concentration of urine OLFM4 as measured by quantitative assay. Bands are present at the expected length of 70 kD for OLFM4.
