# Supplementary material for: Olfactomedin 4 as a novel loop of Henle‐specific acute kidney injury biomarker
Source: Physiol Rep. 2022 Sep 19;10(18):e15453. doi: 10.14814/phy2.15453 (PMC9483618; doi:10.14814/phy2.15453)
Supplement: Supplementary file 2 — Figure S2 [file PHY2-10-e15453-s001.pdf]

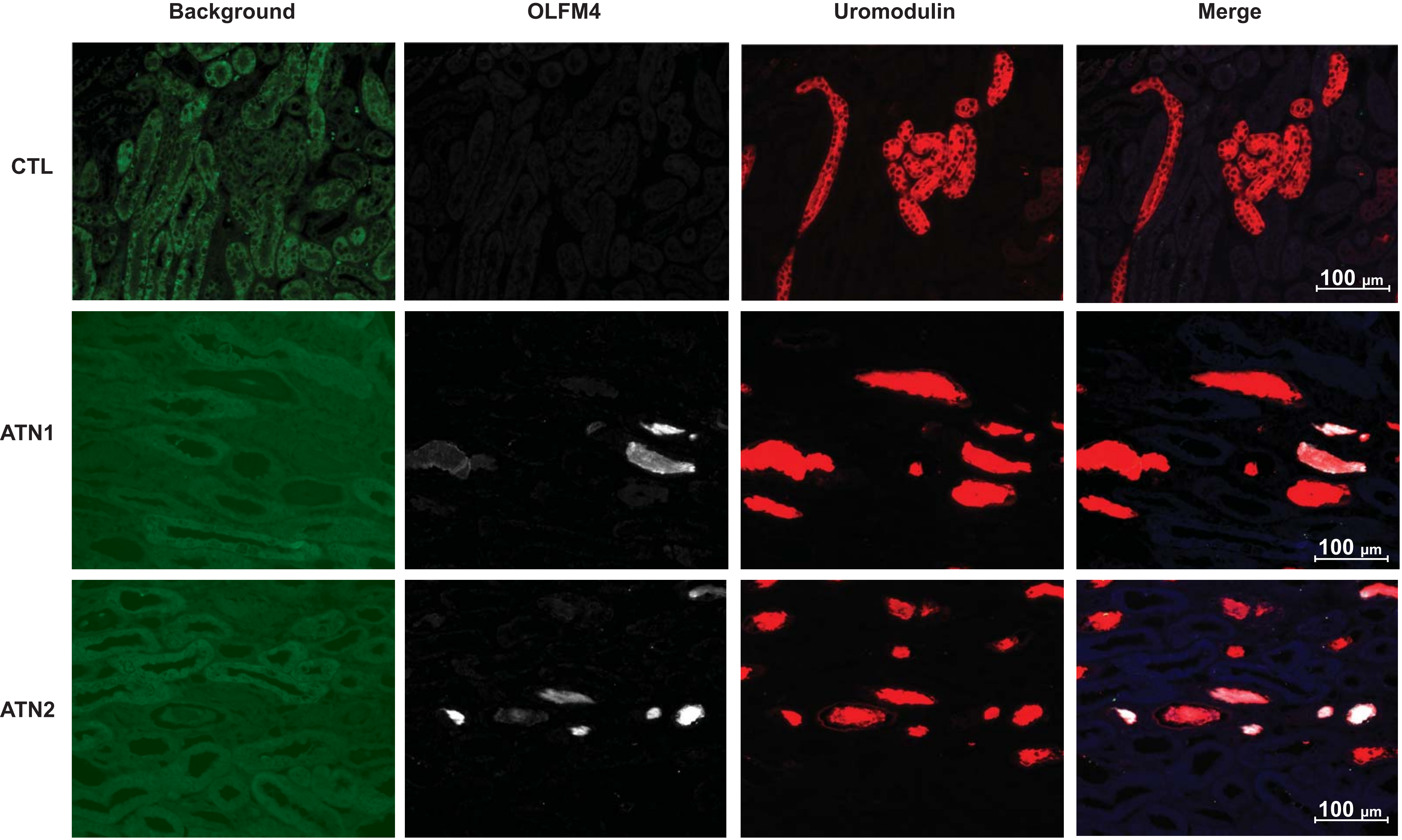

Supplementary Figure 2. Immunofluorescence from 3 human biopsy samples, one control and two with acute tubular necrosis. (Columns left to right). Background shows tubular architecture. OLFM4 staining appears in white. Uromodulin appears in red. Controls non-AKI samples had very rare OLFM4 staining or were devoid of OLFM4 all together. ATN-acute tubular necrosis. OLFM4- olfactomedin 4
